# Calibration between Color Camera and 3D LIDAR Instruments with a Polygonal Planar Board

**DOI:** 10.3390/s140305333

**Published:** 2014-03-17

**Authors:** Yoonsu Park, Seokmin Yun, Chee Sun Won, Kyungeun Cho, Kyhyun Um, Sungdae Sim

**Affiliations:** 1 Department of Electronics and Electrical Engineering, Dongguk University-Seoul, 30 Pildong-ro 1-gil, Jung-gu, Seoul 100-715, Korea; E-Mails: laim4525@dongguk.edu (Y.P.); smyun@dongguk.edu (S.Y.); 2 Department of Multimedia Engineering, Dongguk University-Seoul, 30 Pildong-ro 1-gil, Jung-gu, Seoul 100-715, Korea; E-Mails: cke@dongguk.edu (K.C.); khum@dongguk.edu (K.U.); 3 Agency for Defense Development, Bugyuseong daero 488 beon gi, Yoseong, Daejeon 305-152, Korea; E-Mail: sdsim@add.re.kr

**Keywords:** camera calibration, 3D LIDAR, sensor fusion, calibration board, 3D point clouds, calibration matrix

## Abstract

Calibration between color camera and 3D Light Detection And Ranging (LIDAR) equipment is an essential process for data fusion. The goal of this paper is to improve the calibration accuracy between a camera and a 3D LIDAR. In particular, we are interested in calibrating a low resolution 3D LIDAR with a relatively small number of vertical sensors. Our goal is achieved by employing a new methodology for the calibration board, which exploits 2D-3D correspondences. The 3D corresponding points are estimated from the scanned laser points on the polygonal planar board with adjacent sides. Since the lengths of adjacent sides are known, we can estimate the vertices of the board as a meeting point of two projected sides of the polygonal board. The estimated vertices from the range data and those detected from the color image serve as the corresponding points for the calibration. Experiments using a low-resolution LIDAR with 32 sensors show robust results.

## 1. Introduction

Recently, multi-sensors have been frequently used in the field of robot vision. For instance, a ranging sensor such as high-speed 3D LIDAR is used in conjunction with a color camera for various robot navigation tasks. The 3D LIDAR sensor is capable of providing 3D position and depth information about objects, whereas the color camera captures their 2D color features. Therefore, by providing the 2D image data with the 3D positional information, one can visualize the objects with a more realistic view. However, as a prerequisite, we need to know their relative positions and orientations by calibrating both sensors of the LIDAR and the color camera.

**Figure 1.**
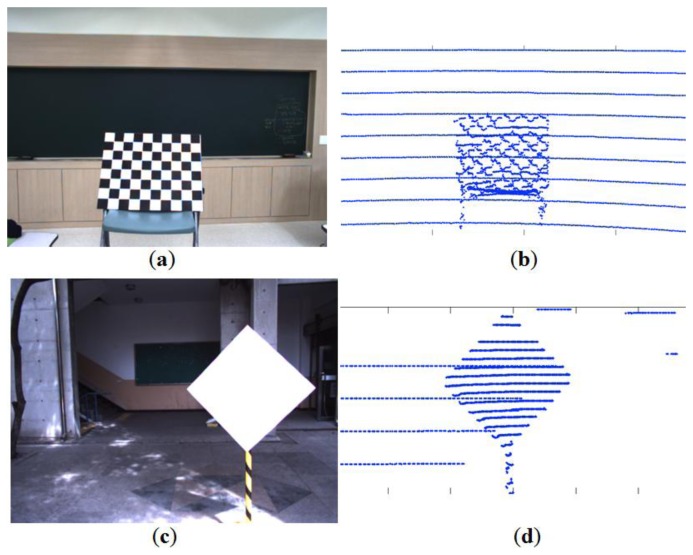
Velodyne HDL-32E scanning on checkerboard and monochromatic board: (**a**) Checkerboard; (**b**) Scanned data of (a); (**c**) Monochromatic board; (**d**) Scanned data of (c).

A checkerboard plane has been used to calibrate between a camera and a LIDAR. The calibration method using a checkerboard usually involves a two-step process [1], namely intrinsic and extrinsic calibrations. Therefore, two measurements from the checkerboard are required for the two-step calibration, which may cause two sources of error [2,3]. Also, we often observe a systematic range-reflectivity-bias in the LIDAR on the checkerboard as seen in Figure 1. The measurement variations on the checkerboard will cause measurements errors and affect the final calibration. Thus, the calibration targets with different patterns and colors may produce slightly different calibration results. To reduce the impact of the reflectivity bias, we use a calibration board with a monochromatic color (e.g., a white planar board). In addition, we adopt a board with a polygonal shape such as triangle or diamond to improve the calibration accuracy. That is, the polygonal board enables us to estimate the vertices (*i.e.*, corners) from the scanned range data. Then, the estimated vertices serve as reference points between the color image and the 3D scanned data for the calibration. The vertices of the polygonal planar board in the 2D image are detected by a corner detection method and their corresponding points in the 3D LIDAR are estimated from the scanned 3D data.

**Figure 2.**
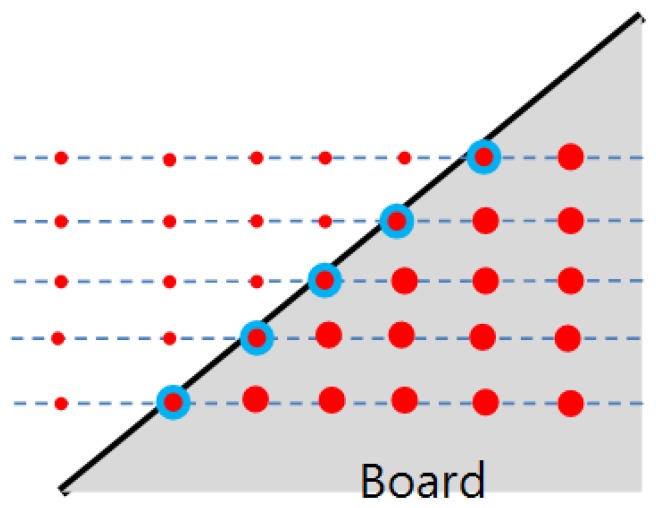
Calibration board with adjacent sides: the scanned points on the border of the plane are used for estimating the side lines of the board.

In this paper we are interested in finding a projection matrix between the camera and the LIDAR directly without needing to perform a separate two-step (*i.e.*, intrinsic and extrinsic) parameter estimation. The direct estimation needs to identify corresponding points between the 2D image and 3D LIDAR to solve the equations for the projection matrix. However, it is hard to expect the LIDAR to scan a specific point such as a vertex of a calibration board, while its corresponding pixel point in the 2D image can be readily detected. For example, a less expensive 3D LIDAR such as Velodyne HDL-32E has a lower vertical resolution compared with a more expensive scanner with 64 sensors such as the Velodyne HDL-64E, making it almost impossible for the 3D LIDAR to scan specific points (e.g., vertices) on the board. With scanners of low vertical resolution, our approach for the direct calibration is to estimate specific unscanned points on the board using the scanned data. That is, given scanned data on the board, we estimate specific 3D positions on the board such as the corners (vertices). To this end, we use a polygonal board, where the vertices of the board are the meeting points between the two adjacent sides. Therefore, to localize the vertices on the planar board we first need to estimate the equations for the projected side lines of the board. As shown in Figure 2, the slope of the projected side can be estimated from the scanned points near the border. Then, with the information of the calculated slopes and the (known) real lengths of the adjacent sides of the planar board, it is possible to calculate their meeting points (*i.e.*, the vertices of the polygonal board).

In this paper, we propose a new calibration method between a camera and a 3D LIDAR using a polygonal board such as a triangle or diamond plane. By estimating the 3D locations of vertices from the scanned laser data and their corresponding corners in the 2D image, our approach for the calibration is to find point-to-point correspondences between the 2D image and the 3D point clouds. The corresponding pairs are used to solve the equations to obtain the calibration matrix.

This paper is composed of the following sections: in Section 2, we survey previous works related to camera and range sensor calibration. The mathematical formulation of the calibration between 2D and 3D sensors is presented in Section 3. In Section 4, we address the proposed calibration method. Experiments conducted on real data are explained in Section 5 and the conclusions follow in Section 6.

## 2. Related Works

Calibration between sensors can be done by finding geometric relationships from co-observable features in the data captured by both sensors. For a color camera and a range sensor, feature points in 2D images can be readily detectable, but it is hard to identify the corresponding 3D points from the range data. Therefore, instead of pinpointing individual 3D feature points, the projected 3D points on the planar board (or on the line) were used to formulate constraints to solve the equations for a transformation matrix. For example, Zhang and Pless [1] proposed the use of a calibration board with a checkerboard pattern on it. Here, the corners of the checker pattern are detected in the images with various board positions for the intrinsic parameter estimation. Then, the estimated intrinsic parameters are used to set a constraint for the extrinsic parameters. Note that if we need the intrinsic parameters, then we take this two-step parameter estimation with the checkerboard. However, if the final goal is to get the projection matrix between the camera and the LIDAR, then it is not necessary to estimate the intrinsic and extrinsic parameters separately. Rather, the two measurements in the separate parameter estimation can cause an additional source of error [2,3].

A planar board plays an important role in the calibration. Wasielewski and Strauss [4] used a rig with a black and white planar board to calibrate a 2D laser scanner with respect to a camera. Willis *et al.* [5] also designed a rig which has many corners that can be used to find the corresponding data in the LIDAR. Naroditsky *et al.* [6] used a white planar board with a black line. In [7], a triangle plane board was used and its side lines were used to minimize the distance between the projected line of the plane and the intersected laser point on the line. Kwak *et al.* [8] also tried to minimize the projected errors of the laser points on the line created by v-shaped planes.

As 3D laser range sensors become popular, the calibration problem turned to a calibration between a 3D LIDAR and a camera [2,3,9–15]. Here, the calibration methods using a planar checkerboard were extended from 2D to 3D LIDAR. Andreasson *et al.* [9] used a calibration board which was framed with a reflective tape, enabling the use of the reflective (remission) data from the laser scanner to automatically estimate the 3D positions of the chess board corners. In [10–12], methods exploiting the detected edges or trihedrons from natural scenes were proposed instead of an extra calibration rig. Lines and corners from indoor or outdoor structured environment were used as reference features for the calibration. Aliakbarpour *et al.* [13] used an Inertial Measurement Unit (IMU) to provide extra information for robust calibration. Also, a simple laser pointer was used as a bright spot to find corresponding points. Pandey *et al.* [14] used three checkerboards which have different normal vectors, because three views are required to completely constrain the six degree of freedom (DOF) pose of one sensor with respect to the other. Geiger *et al.* [15] used multiple sheets of checkerboards. So, the camera and scanner were calibrated using a single image. All the previous methods mentioned above are for the rigidly mounted sensors with off-line calibration. Recently, an on-line calibration for the sensors of occasional movements was proposed [16], where the point-based feature correspondences were used for the calibration.

Many types of special rig for 3D range sensor besides the LIDAR were used to estimate extrinsic parameters between a camera and a range sensor [17–19]. For example, a time-of-flight (ToF) device or Microsoft Kinect™ has a limited field of view compared to the omnidirectional 3D LIDAR, but it can acquire high density 3D point clouds. Jung *et al.* [17] designed a planar board with round holes on it and Shahbazi *et al.* [18] used a multi-resolution white squares pattern on a black plane to calibrate between a camera and the ToF device with a low resolution.

In the previous studies, various types of calibration rigs or environmental structures were used to improve the calibration accuracy. However, the performance of those methods relies on the density and location of actual scanned points on the calibration board (or the environmental structure). This implies that the accuracy of the calibration may drop quickly for a low resolution 3D LIDAR with a relatively small number of sensors. In this work, we solve this problem by adopting the following novel approaches:

(i) We propose a polygonal planar board with adjacent sides as a calibration rig. Then, our calibration matrix can be obtained by simply solving linear equations given by a set of corresponding points between the 2D-3D vertices of the polygonal board.
(ii) The 3D vertices of the polygonal board are estimated, but not measured, from the scanned 3D points on the board. That is, once the geometric structure of the calibration board is known, we can calculate specific 3D points such as the vertices of the board without actually scanning those points. This property enables us to estimate the projection matrix directly using the corresponding pairs between 2D image and 3D points, which is especially useful for a low resolution 3D LIDAR with a relatively small number of sensors.
(iii) Using our approach, the combined projection matrix of the extrinsic and intrinsic matrices can be estimated without estimating them separately. Of course, our method can be used only for the extrinsic transformation matrix as usual.

## 3. Calibration Model for Camera and 3D LIDAR

**Figure 3.**
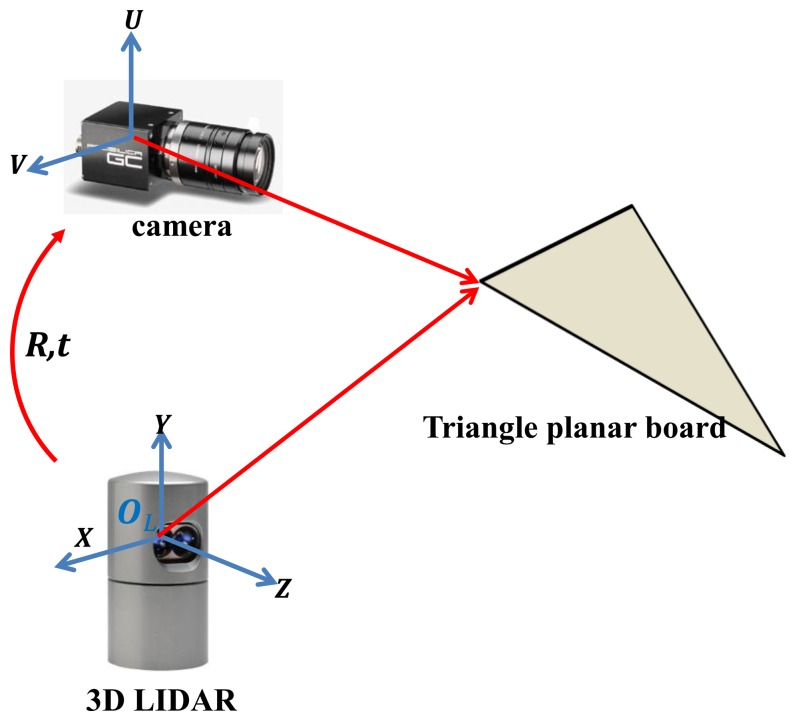
Calibration configuration of a camera and 3D LIDAR with a triangle board.

We set a triangle board in front of the rigidly mounted camera and 3D LIDAR (see Figure 3). A Velodyne HDL-32E LIDAR with 32 vertically mounted laser sensors is used as the 3D LIDAR. The image data captured by the camera are formed by two-dimensional coordinate system (*U*, *V*) and the range data of the 3D point clouds are represented by three-dimensional coordinate system (*X*,*Y*,*Z*). Our goal is to estimate the projective transformation matrix *M* of intrinsic and extrinsic parameters between the color coordinate (*U*, *V*) and the LIDAR coordinate (*X*,*Y*,*Z*). Then the transformation from a 3D point (*x*,*y*,*z*) to a 2D point (*u*,*v*) can be represented by:

(1) $$\left(\begin{array}{c} u\\ v\\ 1 \end{array}\right)=\left(\begin{array}{ccc} f_{u} & 0 & u_{0}\\ 0 & f_{v} & v_{0}\\ 0 & 0 & 1 \end{array}\right)\;\left(\begin{array}{cc} R & t\\ 0 & 1 \end{array}\right)\;\left(\begin{array}{c} x\\ y\\ z\\ 1 \end{array}\right)=M\;\left(\begin{array}{c} x\\ y\\ z\\ 1 \end{array}\right)=\left(\begin{array}{llll} m_{11} & m_{12} & m_{13} & m_{14}\\ m_{21} & m_{22} & m_{23} & m_{24}\\ m_{31} & m_{32} & m_{33} & m_{34} \end{array}\right)\;\left(\begin{array}{c} x\\ y\\ z\\ 1 \end{array}\right)$$

where *f_u_* and *f_v_* are the effective focal lengths in horizontal and vertical directions, respectively, and (*u*_0_, *v*_0_) is the center point of the image plane. Also, *R* and *t* are the rotation and the translation matrices. As one can see in Equation (1), the transformation matrix *M* is a fusion of the intrinsic camera parameters (*f_u_*, *f_v_*, *u*_0_, *v*_0_) and the extrinsic parameters (*R*, *t*) and the matrix coefficient *m_pq_* can be determined by corresponding pairs of (*u*, *v*)and (*x,y,z*). That is, (1) can be rewritten as the following equations:

(2) $$u=\frac{m_{11}x+m_{12}y+m_{13}z+m_{14}}{m_{31}x+m_{32}y+m_{33}z+m_{34}}$$

(3) $$v=\frac{m_{21}x+m_{22}y+m_{23}z+m_{24}}{m_{31}x+m_{32}y+m_{33}z+m_{34}}$$

and in the form of matrix multiplication as:

(4) $$\left(\begin{array}{llllllllllll} x & y & z & 1 & 0 & 0 & 0 & 0 & -ux & -uy & -uz & -u\\ 0 & 0 & 0 & 0 & x & y & z & 1 & -vx & -vy & -vz & -v \end{array}\right)\;\left(\begin{array}{l} m_{11}\\ m_{12}\\ m_{13}\\ m_{14}\\ m_{21}\\ m_{22}\\ m_{23}\\ m_{24}\\ m_{31}\\ m_{32}\\ m_{33}\\ m_{34} \end{array}\right)=\left(\begin{array}{c} 0\\ 0 \end{array}\right)$$

For each corresponding pair we have two equations as in Equation (4). To determine the unknown coefficients *m_pq_* we need a sufficient number of corresponding pairs.

## 4. Vertex Correspondences in Polygonal Board

**Figure 4.**
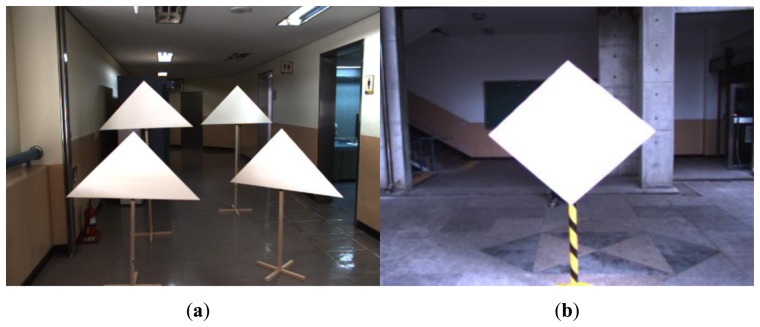
Polygonal planar boards: (**a**) Triangle board and (**b**) Diamond board.

Our calibration method uses a polygonal planar board with adjacent sides (e.g., triangle and diamond boards) (see Figure 4). Li *et al.* [7] also used a triangular board for the calibration, where the reference for the calibration errors in [7] is the boundary line (edge) of the board and the calibration criterion is to minimize the distances from the scanned laser points on the boarder of the plane to the boundary line. In this paper, we use key points (e.g., the vertices) on the board instead of the line to make point-to-point correspondences between 2D image and 3D points, leading direct solution of the linear equations for the estimation of the projection matrix.

Noting that our vertex-based calibration method can be applied for any polygonal board with adjacent sides, we explain our method with a simple triangle planar board and the extension to other polygonal board such as a diamond plane should be straightforward. The overall steps of our method can be summarized as follows.

(i) Data acquisition: Place one or more triangle planar boards in front of the camera and 3D LIDAR. Take camera images and measure the 3D point clouds of the 3D LIDAR for various locations of the board. To reduce the measured errors in the 3D LIDAR and to easily detect vertices of the triangle planar board in the image, it is recommended to use a bright monochromatic color for the board. Also, the board color should be distinctive from the background and the size of the board has to be large enough to include multiple laser scanning lines of the 3D LIDAR on the board surface.
(ii) Matching 2D-3D point correspondences: Detect vertices of the triangle plane in images and identify their corresponding 3D points from the laser scans by estimating the meeting points of two adjacent sides of the board.
(iii) Estimate the calibration parameters between 3D LIDAR and camera. With the corresponding pairs solve the linear equations for the initial estimate and refine the solutions for the final estimates.

Of the above three steps we elaborate steps (ii) and (iii) in the following subsections.

### 4.1. Matching 2D-3D Point Correspondences

In order to solve the linear equations for the transformation matrix, we need to find point-to-point correspondences between the image and the 3D laser point at the vertices of the triangle planar board. For a 2D image, the vertices can be easily detected by a corner detection method such as Features from Accelerated Segment Test (FAST) [20]. Among all the detected corners, only three corners which represent vertices of the triangle plane are selected. The three vertices on the triangle board are located at the top center, *v_iC_*(*u_C_*, *v_C_*), at the lower left, *v_iL_*(*u_L_*, *v_L_*), and at the lower right, *v_iR_*(*u_R_*, *v_R_*). The corresponding vertices in the laser 3D coordinate are *v_C_*(*x_pC_*, *y_pC_*, *z_pC_*), *v_L_*(*x_pL_*, *y_pL_*, *z_pL_*)and *v_R_*(*x_pR_*, *y_pR_*, *z_pR_*). The vertices in the image can be readily detected by the corner detection algorithm, whereas the corresponding vertices in the 3D LIDAR coordinate are hard to locate and the chance to lie the scan line exactly on the three vertices of the triangle board is quite low especially for a low resolution LIDAR such as the Velodyne HDL-32E. In this situation, our strategy to identify the 3D correspondences of the vertices is to estimate them by calculating the meeting points of the side lines on the planar board.

### 4.2. Estimation of 3D Points on the Board

**Figure 5.**
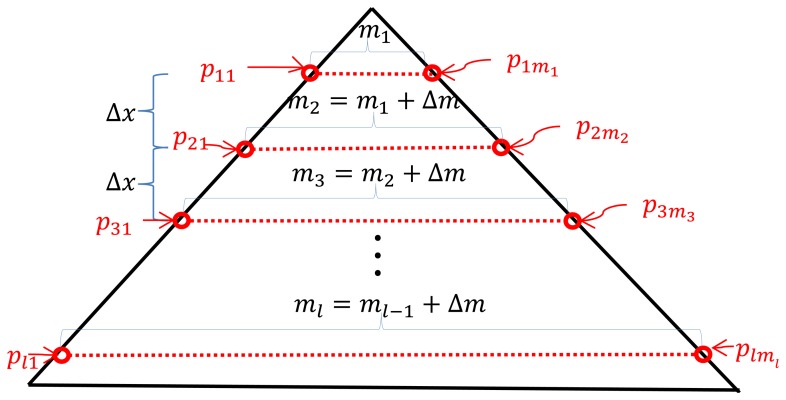
Scanned laser (dotted) lines on the triangle planar board.

**Figure 6.**
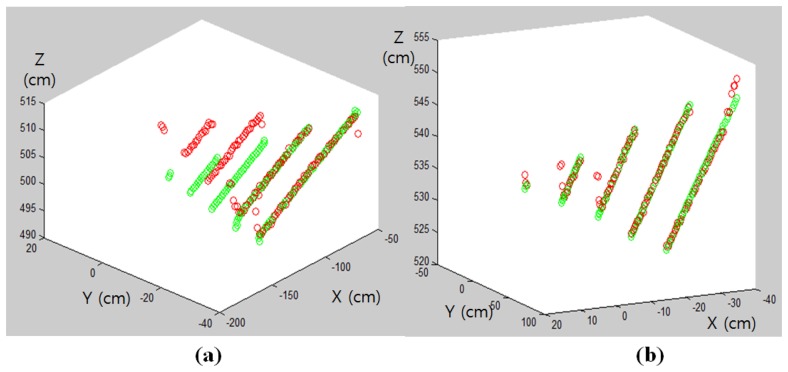
The 3D points (red) and its orthogonal projection (green). The inlier 3D points of the RANSAC are selected by: (**a**) *inlier ratio;* (**b**) *inlier line ratio*.

To locate the vertices on the triangle board in the 3D LIDAR coordinate, we first need to measure the 3D point clouds on the board plane. Suppose that there are *l* scan lines *P* = {*P*_1_, *P*_2_ , … , *P_l_*} on the triangle board and each line *P_n_* at the line *n* consists of *m_n_* points such that *P_n_* = {*P_n_*_1_, *P_n_*_2_ , … , *P_nmn_*}, where *p_nm_* represents the *m*th point in the *n*th scan line on the board scan (see Figure 5). Although the calibration board is a flat panel, the 3D points *P* generated from the 3D LIDAR usually have uneven measurements on the board, so we need to sort out the 3D points which are close to the board surface with smaller errors. To this end, we employ a 3D plane fitting method based on the Random Sample Consensus (RANSAC) [21] algorithm with the following three steps: (i) among all 3D points in *P* we take three sample points at random and calculate the plane equation formed by the points; (ii) according to the calculated plane equation, each 3D point in *P* is classified into either an inlier point or an outlier point by a distance threshold; (iii) repeat the steps (i) and (ii) by selecting another three points randomly in *P* until we find the best fitted plane ***A*** according to the largest *inlier line density*. Here, the *inlier line density* is the density of the 3D points included in the inlier for each line scan on the triangle board. Note that the inlier points selected by the RANSAC algorithm are used to estimate the adjacent sides of the triangle board, which requires the inlier 3D points to be spread all over the scan line. Therefore, we define the *inlier line density* as the criterion of the RANSAC algorithm instead of the total number of inliers, so we first define the *inlier ratio*, which is the ratio between the number of detected inliers and the total number of data as:

(5) $$\text{inlier ratio}=\frac{\sum_{n=1}^{l}I_{n}}{\sum_{n=1}^{l}m_{n}}$$

where *I_n_* is the number of inliers on the scan line *n* and *m_n_* represents the total number of 3D points on the scan line *n*. Note that if we use the *inlier ratio* of Equation (5) for the RANSAC algorithm, then the majority of the inliers are from the bottom lines of the triangle board (see Figure 5) and the plane equation will be biased by the bottom lines. For example, in Figure 6a, red circles represent the input 3D points for the RANSAC algorithm and the green ones are the projected 3D points on the estimated plane. As one can see, the plane estimation is biased by the majority 3D points from the bottom lines of the triangle board and gives large projection errors at the upper scan lines. To solve this problem, we define *inlier line ratio,* where each scan line contributes to the plane estimation equally regardless of the total number of 3D points on each line. This can be done by giving different weights for the points in different scan lines. That is, the weight for the *n*th scan line *w_n_* is inversely proportional to the total number of 3D points on the line:

(6) $$w_{n}=\frac{1}{m_{1}+(n-1)\Delta m}$$

where we assume that the vertical distance Δ*x* between two consecutive scan lines and their 3D point increment Δ*m* are constant (see Figure 5). That is, the number of 3D points at line *n* can be represented by an arithmetic series *m_n_* =*m*_1_ + (*n* − 1)Δ*m* and *w_n_* in Equation (6) is inversely proportional to the total number of 3D points at each scan line. Multiplying *m_n_* and *I_n_* by *w_n_*, our *inlier line ratio* is defined by:

(7) $$\text{inlier line ratio}=\frac{\sum_{n=1}^{l}w_{n}I_{n}}{\sum_{n=1}^{l}w_{n}m_{n}}$$

By using the *inlier line ratio* in Equation (7), all scanning lines contribute equally regardless of their lengths and we can avoid the bias problem of the *inlier ratio*. For example, using the *inlier line ratio* we can obtain more accurate plane estimation as shown in Figure 6b.

**Figure 7.**
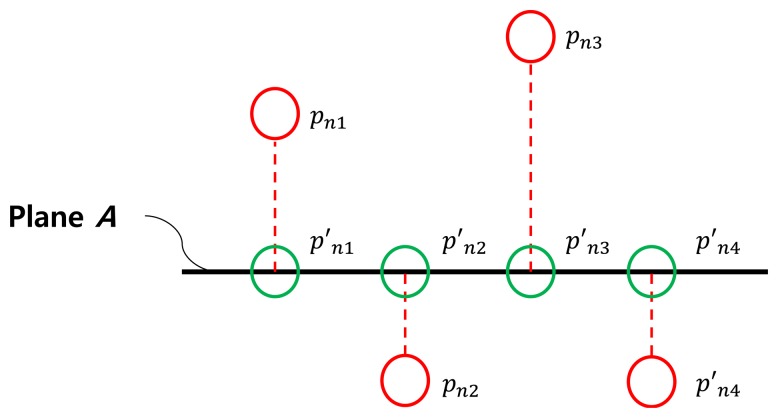
Projection of 3D points (red) *P* onto the estimated plane *A* represented by green circles *P*′.

Once we estimate the board plane using the inlier 3D points of the RANSAC algorithm, we can project all the scanned 3D points *P* onto the estimated plane to have the projected 3D points $P\prime =\left\{P_{1}\prime ,P_{2}\prime ,\ldots ,P_{l}\prime \right\}$, where $P_{n}\prime =\left\{p\prime_{n1},p\prime_{n2},\ldots ,p\prime_{1m_{1}}\right\}$(see Figure 7).

### 4.3. Estimation of Vertices in Triangle Board

To estimate the three vertices of the triangle planar board in the LIDAR coordinate we use the projected 3D points *P*′ on the estimated board plane *A*. To this end, we estimate the slopes of the side lines in the triangle planar board. Then, we can determine the 3D positions of the vertices by calculating the meeting points of two side lines.

**Figure 8.**
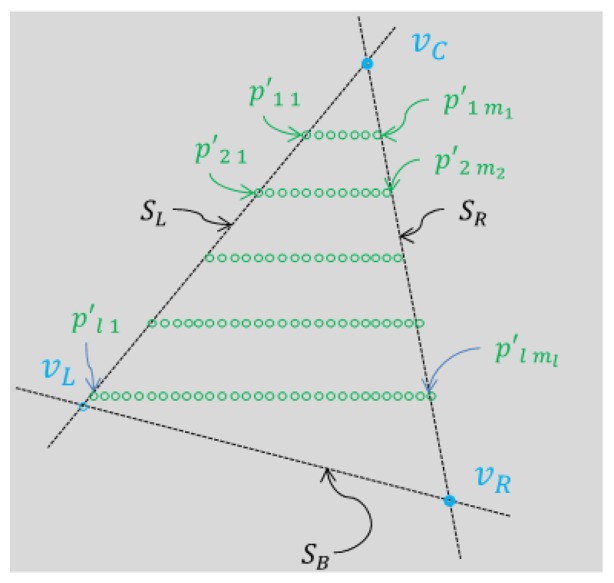
Vertices, adjacent lines, and projected points on the triangle board.

Let us denote the three sides of the triangle board as *S_L_* for the left side, *S_R_* for the right side, and *S_B_* for the bottom side (see Figure 8). Also, we denote the segments of each side as $\bar{S_{L}}$, $\bar{S_{R}}$, and $\bar{S_{B}}$ in the 3D LIDAR coordinate. The straight lines which include $\bar{S_{L}}$, $\bar{S_{R}}$, and $\bar{S_{B}}$ are expressed as $\overset{↔}{S_{L}}$, $\overset{↔}{S_{R}}$, and $\overset{↔}{S_{B}}$ and the vectors representing the slopes of $\overset{↔}{S_{L}}$, $\overset{↔}{S_{R}}$, and $\overset{↔}{S_{B}}$ are $\vec{S_{L}}=\left[S_{Lx},S_{Ly},S_{Lz}\right]$, $\vec{S_{R}}=\left[S_{Rx},S_{Ry},S_{Rz}\right]$, and $\vec{S_{B}}=\left[S_{Bx},S_{By},S_{Bz}\right]$, respectively.

**Figure 9.**
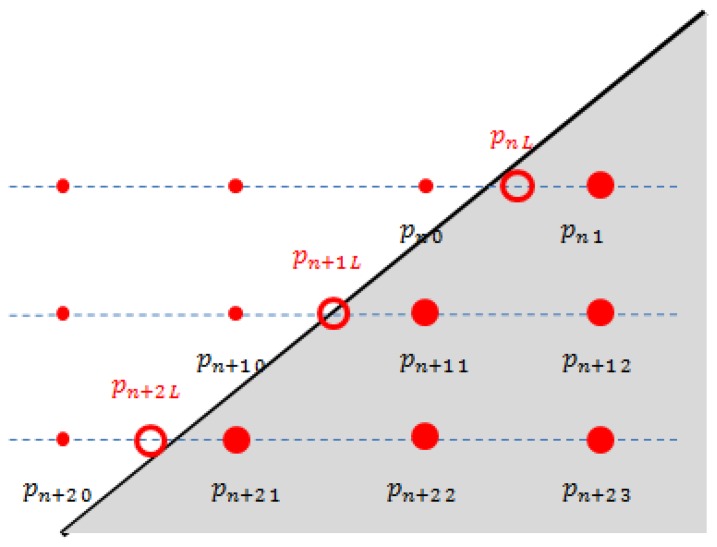
Virtual points (empty circles) near the side line.

To calculate $\overset{↔}{S_{L}}$ and $\overset{↔}{S_{R}}$, a 3D line fitting method based on the RANSAC algorithm is used. That is, the estimation of the side line is based on the projected 3D points near the border of the triangle plane, namely { $p\prime_{11}$, $p\prime_{21}$, …, $p\prime_{l1}$} for the left line and { $p\prime_{1m_{1}}$, $p\prime_{2m_{2}}$, …, $p\prime_{lm_{l}}$} for the right line (see Figure 8). Here, to improve the accuracy of the line estimation, we can use the virtual points between the two consecutive points, where one is off the board and the other is on the board, e.g., *p_n_*_0_ and *p_n_*_1_ in Figure 9. Specifically, the virtual point $p\prime_{nL}$ is between *p_n_*_0_ and *p_n_*_1_. Also, $p\prime_{nR}$ is between *p_nmn_* and *p_nm_*_1_+1. The locations of the virtual points are determined by the average distance between the scanned points for each scan line. So, by calculating the average Euclidean distance $\Delta d_{n}=\text{dist}(p\prime_{n1},p\prime_{nm_{n}})/m_{n}$ for the scan line *n*, we can locate the virtual 3D points on the left and the right sides as follows:

(8) $$\begin{array}{c} {p^{\prime }}_{nL}=p\prime_{n1}-\frac{\Delta d_{n}}{2}\vec{\left|p\prime_{nm_{n}}-p\prime_{n1}\right|}\\ {p^{\prime }}_{nR}=p\prime_{nm_{n}}+\frac{\Delta d_{n}}{2}\vec{\left|p\prime_{nm_{n}}-p\prime_{n1}\right|} \end{array}$$

Now, locating the boundary points $P\prime_{L}=\left\{p\prime_{1L},p\prime_{2L},\ldots ,p\prime_{lL}\right\}$ and $P\prime_{R}=\left\{p\prime_{1R},p\prime_{2R},\ldots ,p\prime_{lR}\right\}$, we can estimate $\overset{↔}{S_{L}}$ and $\overset{↔}{S_{R}}$, respectively, as follows:

(9) $$\begin{array}{c} \overset{↔}{S_{L}}:\frac{x-x_{nL}}{S_{Lx}}=\frac{y-y_{nL}}{S_{Ly}}=\frac{z-z_{nL}}{S_{Lz}}\\ \overset{↔}{S_{R}}:\frac{x-x_{nR}}{S_{Rx}}=\frac{y-y_{nR}}{S_{Ry}}=\frac{z-z_{nR}}{S_{Rz}} \end{array}$$

where $p\prime_{nL}=\left(x_{nL},y_{nL},z_{nL}\right)$ and $p\prime_{nR}=\left(x_{nR},y_{nR},z_{nR}\right)$ are the 3D coordinates of the virtual points on the projected scan line *n*. Also, $\vec{S_{L}}=\left[S_{Lx},S_{Ly},S_{Lz}\right]$ and $\vec{S_{R}}=\left[S_{Rx},S_{Ry},S_{Rz}\right]$ denote the slopes of the left and the right side lines.

**Figure 10.**
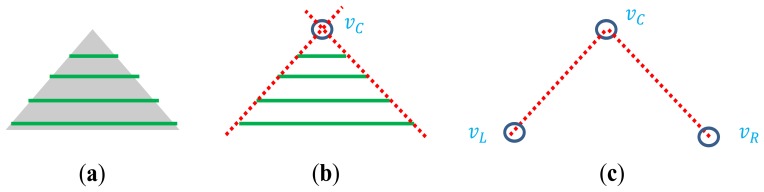
Vertex estimation process for the triangle board: (**a**) Projection of 3D points on the plane ***A***.; (**b**) Detection of the center vertex as the meeting point of the adjacent sides; (**c**) Estimation of the left and right vertices from the known lengths of adjacent sides.

The 3D coordinate of the center vertex on the triangle *v_C_* can be detected by finding the intersection of $\overset{↔}{S_{L}}$ and $\overset{↔}{S_{R}}$. Since $\overset{↔}{S_{L}}$ and $\overset{↔}{S_{R}}$ are generated from the projected 3D points *P*′ on the plane ***A***, there always exists an intersection of the two lines. The intersection of $\overset{↔}{S_{L}}$ and $\overset{↔}{S_{R}}$ is the top vertex *v_C_* on the triangle plane. Once we identify the 3D coordinate of the top vertex *v_C_*, we can calculate the 3D coordinates of the other two vertices *v_L_* and *v_R_* by using the known lengths of the side lines ${\left|\bar{S_{L}}\right|}_{\text{real}}$ and ${\left|\bar{S_{R}}\right|}_{\text{real}}$ as follows (see Figure 10):

(10) $$\begin{array}{c} v_{L}=v_{C}-{\left|\bar{S_{L}}\right|}_{\text{real}}\vec{S_{L}}\\ v_{R}=v_{C}-{\left|\bar{S_{R}}\right|}_{\text{real}}\vec{S_{R}} \end{array}$$

### 4.4. Suitability Test for the Detected Vertices

The suitability of the detected vertices can be tested by comparing the known real length ${\left|\bar{S_{B}}\right|}_{\text{real}}$ of the bottom line of the triangle and its estimated length $\left|\bar{S_{B}}\right|=\left|v_{L}-v_{R}\right|$ from the detected vertices *v_L_* and *v_R_*. That is, the following normalized error *ε_B_* between ${\left|\bar{S_{B}}\right|}_{\text{real}}$ and $\left|\bar{S_{B}}\right|$ is used to test the suitability of the vertex estimation:

(11) $${ɛ}_{B}=\left|\frac{{\left|\bar{S_{B}}\right|}_{\text{real}}-\left|\bar{S_{B}}\right|}{{\left|\bar{S_{B}}\right|}_{\text{real}}}\right|$$

If *ε_B_* in Equation (11) is less than a threshold *T_B_*, then we declare that the estimated vertices *v_C_*, *v_L_*, and *v_R_* are accurate enough and accept them as the coordinates of the vertices. Otherwise, we go back to the first step of the plane estimation.

### 4.5. Estimation of Calibration Matrix

The vertices of the triangle board captured by the camera as a 2D image can be readily detected by a corner detection method such as FAST [20]. Then, we have *n* pairs of vertices of the polygonal boards between the 3D points ℙ_3_ = {(*x*_1_, *y*_1_, *z*_1_),( *x*_2_, *y*_2_, *z*_2_), … , ( *x_n_*, *y_n_*, *z_n_*)} and their corresponding 2D points ℙ_2_ = {(*u*_1_,*v*_1_), (*u*_2_,*v*_2_), … , (*u_n_*, *v_n_*)}, where (*x_i_*, *y_i_*, *z_i_*) and (*u_i_*, *v_i_*) are the 3D and 2D coordinates, respectively, for a vertex on the polygonal planar board. Given these *n* pairs of correspondences, we have 2*n* linear equations by substituting each correspondence to Equation (4). Then, by using the singular value decomposition (SVD) method, we can solve the linear equations. However, due to some measurement errors of the correspondence pairs, the solution of the projection matrix does not yield an exact transformation matrix. Therefore, we need a refinement process such that, starting from the solution of Equation (4), we iteratively update the solution by using a nonlinear least squares method. Specifically, Levenberg-Marquardt algorithm [22] can be applied to update the solution of Equation (4) for the final solution.

### 4.6. Extension to a Diamond-Shape Planar Board

**Figure 11.**
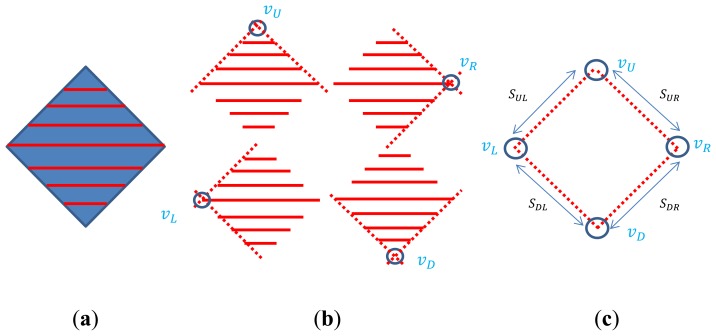
Diamond board with four vertices. (**a**) Scan lines on the board; (**b**) Vertex detection as an intersection of two adjacent sides; (**c**) Suitability test by accumulated errors between the real (known) length and estimated one.

Note that, as we have more scan lines on the board, we can estimate the plane more accurately. Also, a polygonal structure with more intersections between edges definitely improves the accuracy of the solution for the camera calibration. For example, a diamond board with four vertices as in Figure 11 should be better than a triangle board. The vertex detection method for the triangle board can be directly applied to the diamond board. That is, in the diamond board we can estimate each vertex by computing the intersection of the adjacent side lines. Then, the suitability test for the detected vertices can be done by accumulating the distance errors between the known real length of the side line and its estimated length from the detected vertices. Specifically, the normalized error in Equation (11) is accumulated for each line in the diamond and tested by a threshold *T_B_*.

## 5. Experimental Results

**Figure 12.**
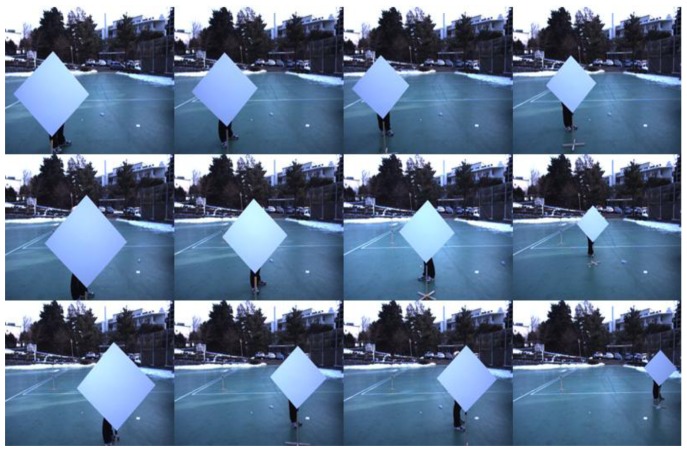
Diamond boards with 12 different positions: the distances from the camera to the board are 1.7 m, 2.2 m, 3 m and 5∼7 m.

Experiments with the diamond planar board are conducted to evaluate the performance of our method. The lengths of four sides of the diamond board used in our experiment are known and equal to 72 cm. For the sensors we used a color camera with resolution of 659 × 493 and a Velodyne HDL-32E LIDAR (see Figures 3). The Velodyne HDL-32E LIDAR has 360° horizontal and 41.3° (+10.67 to −30.67) vertical field of view with 32 scan lines, so its vertical angular resolution is 1.33 degree. With these sensors we took 2D images and 3D LIDAR data with 12 different positions of the diamond board as shown in Figure 12.

**Figure 13.**
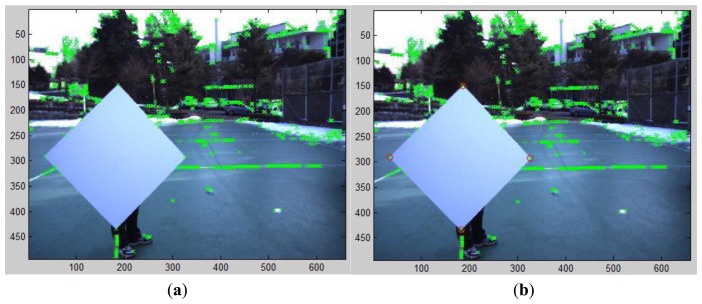
Selection of four corners on the diamond board in 2D image: (**a**) Detected corners in the image with FAST method (green cross markers); (**b**) Selected 4 corners on the diamond board (red circle markers).

**Figure 14.**
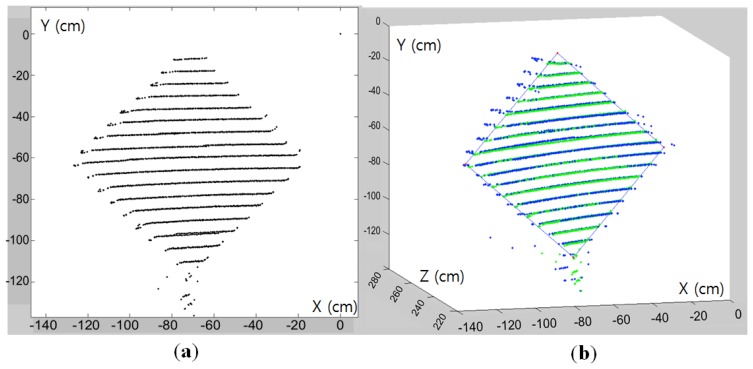
Lasers scans on the diamond board: (**a**) 3D points on the diamond board surface; (**b**) estimated side lines and their intersections (red dots) as estimated 3D corners.

Our correspondence-based estimation of *M* in Equation (1) can be applied to 2D images with or without compensating lens distortion. In our experiments, we used the 2D image data without compensating the lens distortion. To find the correspondences of the vertices between the 2D image and the 3D laser data, we first detect 4 corners on the diamond board in the image. As shown in Figure 13, these corners are selected from the detected key points of the FAST algorithm. Once all key points including the four vertices are detected by the FAST algorithm, the exact locations of the vertices are determined by clicking the mouse around the detected vertices manually. Therefore, the role of the FAST algorithm is to locate the exact 2D coordinates of the vertices which can be easily selected by mouse clicking near the point. The corresponding corners in the 3D coordinate of the LIDAR are estimated from the side lines of the estimated plane with a threshold *T_B_* = 0.01 in Equation (11) (see Figure 14 for the estimated corners on the diamond board).

Now, we have four corresponding corners between the 2D image and 3D data and are ready to solve the equations for the projection matrix. Note that we need more than 12 correspondence pairs for estimating 12 calibration parameters and we have to take more than three different positions of the diamond board. Then, the calibration parameters are determined by solving the linear equations and the refinement process.

**Figure 15.**
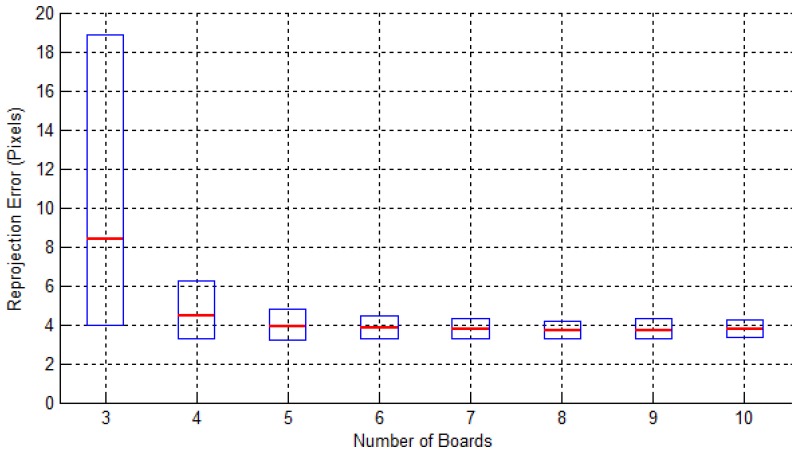
Box-plots of reprojection (pixel) errors for different numbers and positions of the diamond board. The red line in the boxes represents the average error and the extents of the boxes are at 25th and 75th percentiles.

To evaluate the accuracy of the proposed method for different positions of the diamond board, we executed our calibration method for various positions of the diamond board and calculated the calibration pixel errors. Among all 12 positions in Figure 12, we select three positions for the calibration. Then, we have a total of _12_C_3_ = 220 possible combinations for the experiments. For each experiment we have 3 × 4 = 12 corresponding vertex pairs for the solution of the matrix equation. Once we have the final estimation of the calibration matrix, we can compute the reprojection errors for all 48 vertices in all 12 positions. The reprojection errors are calculated based on the distances in pixels between the vertex in 2D and its projected 3D vertex by the estimated matrix. Then, we calculate the average root mean squares for all 48 reprojection errors. The results are shown as box-plots in Figure 15. As one can see from the results, the reprojection errors decrease as the number of boards used increases and they sharply drop after three to four boards. The mean values of the reprojection errors converged to about 4 pixels after five board sets.

**Figure 16.**
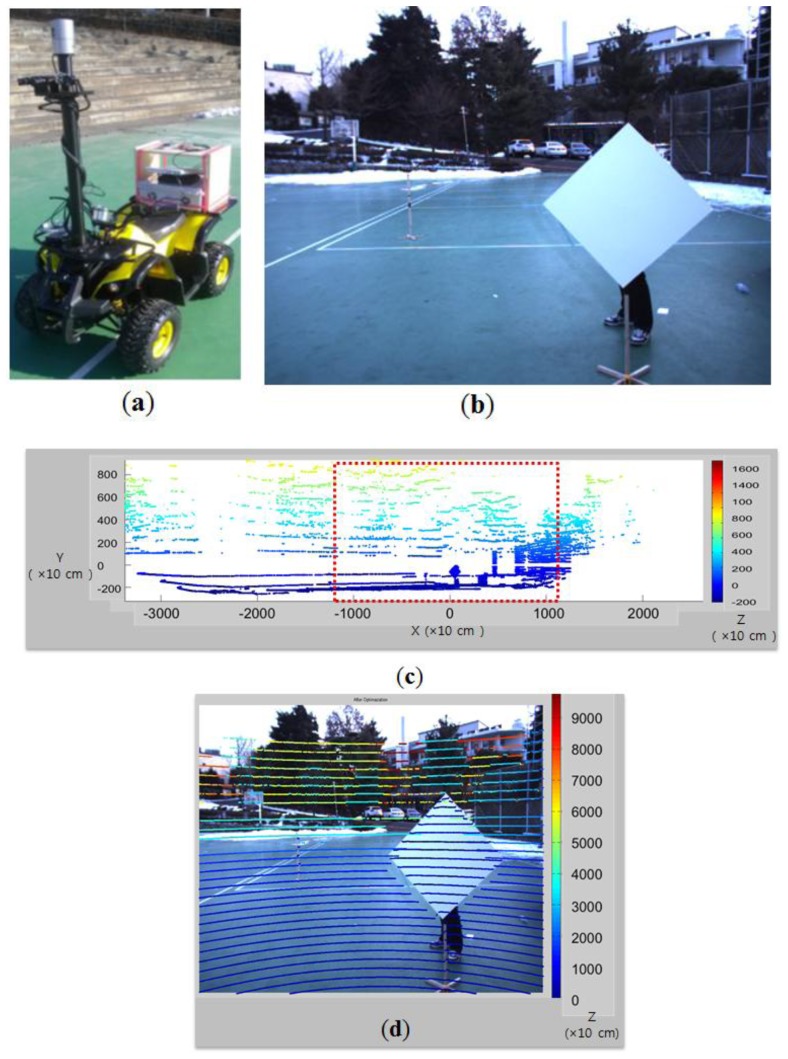
Composition of 3D laser data on the color image by the estimated calibration matrix. (**a**) Cart equipped with camera and Velodyne HDL-32E LIDAR; (**b**) Diamond shaped calibration board; (**c**) 3D point clouds; (**d**) Superimposed color image with the calibrated 3D point clouds (depths are represented by colors on the scan lines).

After the calibration of the camera and the LIDAR (see Figure 16a), we superimpose the 3D laser data on the 2D image according to the estimated projection matrix. The result is shown in Figure 16, where on the 2D image of Figure 16b the 3D laser data of Figure 16c in the red-dot box of the camera's field of view are superimposed. As one can see in Figure 16d, the superimposed 3D data match the actual depths of the 2D image quite well.

**Figure 17.**
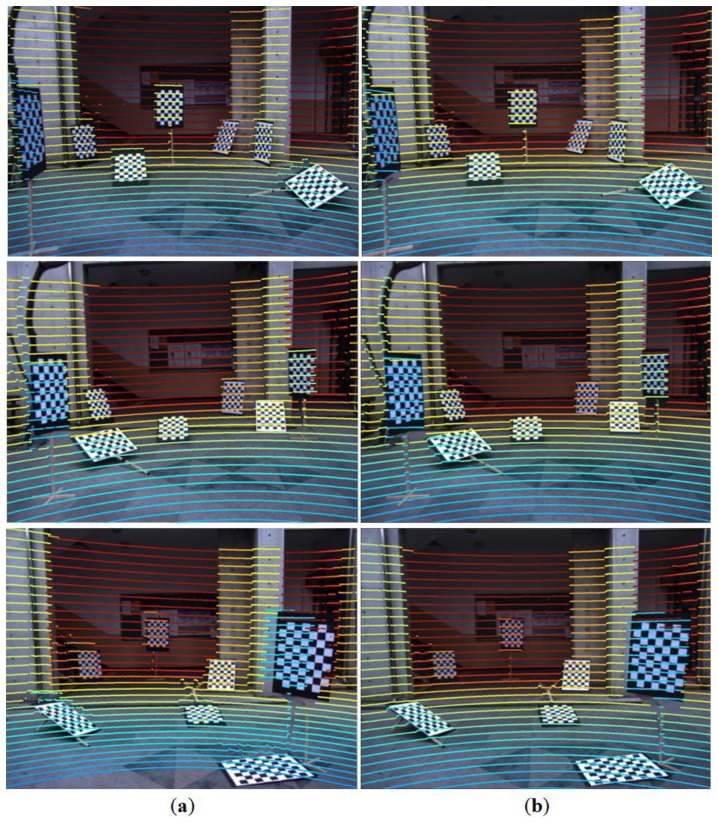
Comparative results: (**a**) Checkerboard method of [15]. (**b**) The projection matrix is estimated by the proposed method; Then, the estimated projection matrix is used to reproject the 3D data of the checkerboard of (**a**) for visual comparison (depths are represented by colors on the scan lines).

We conducted comparative experiments with the checkerboard method in [15]. The estimated parameters using [15] are used to reproject the 3D scan data on the 2D checker image as in Figure 17a. Also, we applied our method to estimate the projection matrix. Then, as in Figure 17b, the projection matrix is used to reproject the 3D scan data of the checkerboard onto the 2D checker image to facilitate the visual comparisons (*i.e.*, the 2D images and 3D scan data from diamond calibration boards are used only for the parameter estimation not for the reprojection). Overall, from Figure 17, we can notice that our method represents the depths on the boundaries of the objects more accurately (e.g., see the results at the third row (bottom)).

## 6. Conclusions

In this paper, we have proposed a new approach for the calibration of a camera and a 3D LIDAR based on 2D-3D key point correspondences. The corresponding 3D points are the vertices of a planar board with adjacent sides and they are estimated from the projected 3D laser points on the planar board. Since our approach is based on 2D-3D point correspondences, the projection matrix can be estimated without separating the intrinsic and extrinsic parameters. Also, our monochromatic calibration board provides more reliable measurements of the 3D points on the board than the checkerboard. Experimental results confirm that our 2D-3D correspondence based calibration method yields accurate calibration, even for a low resolution 3D LIDAR.

## References

[1] Zhang Q., Pless R. Extrinsic calibration of a camera and laser range finder (improves camera calibration).

[2] Yang H., Liu X., Patras I. A simple and effective extrinsic calibration method of a camera and a single line scanning lidar.

[3] Zhou L., Deng Z. A new algorithm for computing the projection matrix between a LIDAR and a camera based on line correspondences.

[4] Wasielewski S., Strauss O. Calibration of a multi-sensor system laser rangefinder/camera.

[5] Willis A.R., Zapata M.J., Conrad J.M. A linear method for calibrating LIDAR-and-camera systems.

[6] Naroditsky O., Patterson A., Danilidis K. Automatic alignment of a camera with a line scan LIDAR system.

[7] Li G., Liu Y., Dong L., Cai X. An algorithm for extrinsic parameters calibration of a camera and a laser range finder using line features.

[8] Kwak K., Huber D.F., Badino H., Kanade T. Extrinsic calibration of a single line scanning lidar and a camera. In Intelligent Robots and Systems (IROS).

[9] Andreasson H., Lilienthal A. (2010). 6D scan registration using depth-interpolated local image features. Robot. Autonom. Syst..

[10] Kern F. (2002). Supplementing lasers canner geometric data with photogrammetric images for modeling. ISPRS.

[11] Scarmuzza D., Harati A., Siegwart R. Extrinsic self calibration of a camera and a 3d laser range finder from natural scenes.

[12] Gong X., Lin Y., Liu J. (2013). 3D LIDAR-camera extrinsic calibration using an arbitrary trihedron. Sensors.

[13] Aliakbarpour H., Núñez P., Prado J., Khoshhal K. An efficient algorithm for extrinsic calibration between a 3d laser range finder and a stereo camera for surveillance.

[14] Pandey G., McBride J., Savarese S., Eustice R. Extrinsic calibration of a 3d laser scanner and an omnidirectional camera.

[15] Geiger A., Moosmann F., car O., Schuster B. Automatic camera and range sensor calibration using a single shot.

[16] Levinson J., Thrun S. Automatic online calibration of cameras and lasers.

[17] Jung J., Jeong Y., Park J., Ha H. A novel 2.5 D pattern for extrinsic calibration of tof and camera fusion system.

[18] Shahbazi M., Homayouni S., Saadatseresht M., Sattari M. (2011). Range camera self-calibration based on integrated bundle adjustment via joint setup with a 2D digital camera. Sensors.

[19] Herrera D., Kannala J., Heikkilä J. (2011). Accurate and Practical Calibration of a Depth and Color Camera Pair. Computer Analysis of Images and Patterns.

[20] Rosten E., Drummond T. (2006). Machine Learning for High-Speed Corner Detection. Computer Vision–ECCV.

[21] Fischler A., Bolles C. (1981). Random sample consensus: A paradigm for model fitting with applications to image analysis and automated cartography. Commun. ACM.

[22] Moré J. (1978). The Levenberg-Marquardt Algorithm: Implementation and Theory. Numerical Analysis.

